# Editorial: Challenging the Functional Connectivity Disruption in Neurodegenerative Diseases: New Therapeutic Perspectives Through Non-invasive Neuromodulation and Cutting-Edge Technologies

**DOI:** 10.3389/fnins.2018.00554

**Published:** 2018-08-08

**Authors:** Giancarlo Zito, Takashi Hanakawa

**Affiliations:** ^1^Unit of Neurology, Ospedale San Giovanni Calibita Fatebenefratelli, Rome, Italy; ^2^Innovation and Pharmaceutical Strategy Division, Italian Medicines Agency, Rome, Italy; ^3^Department of Advanced Neuroimaging, Integrative Brain Imaging Center, National Center of Neurology and Psychiatry, Tokyo, Japan

**Keywords:** rehabilitation, Biorobotics, Translational research, Neurophysiology, neuroprostheses, virtual reality environment, non-invasive brain stimulation, personalized treatment

A growing body of work indicates that in several acute and chronic neurological conditions, including stroke and neurodegenerative diseases, pathophysiological events may introduce functional alterations over small- and large-scale functional network dynamics. These phenomena occur ever since, or even before, the appearance of the first, distinct symptoms, and involve brain connectivity under either a resting or active task condition. The alterations of network dynamics can affect the outcome of subsequent therapeutic interventions and re-learning mechanisms during rehabilitation (Krakauer, 2006). Hence, an in-depth knowledge of early predictors of such network changes is crucial to address the most effective therapeutic and rehabilitation strategies, and to help reduce the burden on neurological patients and health care systems. To disentangle how changes of network dynamics take place in the brain, several functional domains, including motor, sensory, and cognitive ones, should be considered. It is also important to parse not only the extent and distribution of network damage across different conditions, but also to implement cutting-edge methods that are able to integrate specific information of networks on a broader perspective (Pievani et al., 2014). In this research topic, we will see how investigators highlighted the value of continuous and mutual osmosis between clinical research and technology, such as bio-robotics, robot-aided rehabilitation, non-invasive neurostimulation, neuroimaging, and neuroengineering, which can give rise to innovations in the field of neurorehabilitation.

## Monitoring effects of intervention or disease-related brain states

The combination of different neuroscientific techniques provides new opportunities for researchers to address new questions. Das et al. developed a mouse model to study neuroplasticity induced by transcranial direct current stimulation (tDCS) and showed polarity- and genetics-dependent effects of anodal tDCS-induced decreases in vestibulo-ocular reflex gain after cerebellar stimulation. Seewoo et al. explored possible ways to translate knowledge about the actions of repetitive transcranial magnetic stimulation (rTMS) from pre-clinical research in rodents to clinically plausible neuromodulation techniques in humans. In particular, they indicate that the combination of rTMS with fMRI requires specific adjustments to experimental protocols to efficiently scale and interchange paradigms borrowed from rTMS in rodents to fMRI in humans, and vice versa. Neuromodulation with simultaneous electroencephalography (EEG), functional MRI (fMRI), or positron emission tomography (PET) would shed light on a deeper characterization of the complexities behind connectivity dynamics induced by therapeutic or neuromodulatory rTMS.

Yamasaki et al. discussed the “connectopathy,” which might underlie multifaceted deficits in social and behavioral domains in children with autistic spectrum disorders (ASD). Their contribution focused on how visual-evoked and event-related potentials (VEP/ERP) and diffusion tensor imaging (DTI) might reveal tiny alterations of visual and attentional networks in this condition. The ASD “connectopathy” likely arises from abnormal functional and structural connectivity in cortical networks involved in social information processing.

Machine learning methods are being extensively used for extracting neurophysiological features allowing individual categorization of patients with many neurologic conditions. Ogata et al. successfully trained a Support Vector Machine (SVM) on resting-state fMRI images to isolate specific functional connectivity features in idiopathic normal pressure hydrocephalus (iNPH). Moreover, the SVM was able to grade severity of the classical triad in iNPH (cognitive, gait, and urinary disturbances) using resting-state connectivity.

## Virtual reality and robotics

Over the past 25 years, robotics and virtual reality have been increasingly used either to measure human movement or to gain knowledge on how the brain learns and controls voluntary movement (Reinkensmeyer et al., 2004). Robot-aided paradigms enable participants to conveniently tune several physical factors involved in movement simultaneously, thus allowing researchers to examine realistic motor cortex physiology, while minimizing mental frustration and physical harm in patients with motor dysfunctions (Woldag and Hummelsheim, 2002). Despite the great progress in the field of both computer science and engineering, however, much can be done to improve the contribution of virtual reality and robotics to the strategies behind functional recovery of damaged nervous functions (Holden, 2005). Marchal-Crespo et al. introduced fMRI-compatible robot-aided training strategies in a complex locomotor learning task—involving the coordination of the legs in a gait-like pattern to track a Lissajous figure presented on a screen, during fMRI. This is a nice experiment that showed the existence of a mathematical relationship between the error reduction induced by training and the initial skill level, with an error-amplification dependent engagement of cerebral reward circuits. Benyoucef et al. proposed highly realistic virtual simulations to study mental disorders. Although the concept needs to be proved, virtual reality could enhance active participation of patients with mental disorders in experimental studies, overcoming intrinsic communicative, emotional, and affective barriers.

## Physiological and pharmacological neuromodulation for neurological conditions

Pinto et al. discussed in detail how pharmacological interventions with selective serotonin reuptake inhibitors (SSRI), which are widely used for the treatment of depression, may influence recovery after stroke.

Along with pharmacological methodology, non-invasive brain stimulation has been extensively studied in many neuro-psychiatric conditions as a physiological method to induce changes across regions of the nervous system. Quartarone et al. discusses the therapeutic potential of rTMS and tDCS and their neurophysiologic effects (“after effects”) in dystonia. They also pointed out the limitation of the current techniques due to high variability of the outcomes. It is thus necessary to gather all the relevant information from overwhelmingly heterogeneous study protocols and then to convert them into a more harmonized protocol, which would make the interpretation of different studies easier. In this regard, Marceglia et al. proposed a new protocol for focal hand dystonia, using cathodal tDCS bilaterally over the primary motor cortex for 20 min daily for 5 consecutive days. Focal hand dystonia is a movement disorder that can be highly disabling since it often affects body parts essential for professional activity such as the fingers and wrists in pianists. tDCS seems to be a potentially effective non-invasive approach to revert cortical excitability to the normal state, thereby improving dystonic symptoms.

The damage induced by inflammation in multiple sclerosis (MS) represents another important model of functional disruption of networks. MS is a pathological entity in which inflammatory demyelination within the central nervous system leads to an ever-increasing depletion of synaptic transmission and effective plasticity due to a dissemination of lesions in both time and space. For unknown reasons, imaging and clinical findings are not well correlated in MS (known as “clinico-radiological paradox”). This morpho-functional disconnection prevents clinicians from formulating an accurate prognostic judgment about recovery. Stampanoni Bassi et al. give an overview of several non-invasive techniques used in the study of MS, with the aim of clarifying how the choice of targeted brain areas influences the evaluation for the extent of recovery, while providing a holistic organizational view of brain networks in a comprehensive biochemical and neurophysiologic perspective. They also rehearse the interesting concept of diaschisis and extend it to the functional connectome (Carrera and Tononi, 2014) to explain the paradox.

It is important to be aware of the individual variations of the brain anatomy undergoing stimulation. Cancelli et al. extended their technology for personalizing neuromodulatory intervention using transcranial electrical stimulation (tES), aiming at better fitting the electrodes' shape over the cerebral cortical foldings. Their innovative technique achieved individually tailored electrode positioning without a neuronavigation system. This development may allow patients to undergo convenient, yet accurate and affordable tES treatment at home in the future.

Moving toward a better comprehension of mechanisms underlying gait impairment in Parkinson's disease (PD)-the most prevalent movement disorder-Cai et al. applied sinusoidal or stochastic galvanic vestibular stimulation (GVS) to a small group of patients with PD. GVS was able to enhance the functional connectivity between cortical/subcortical regions of interests and the putative pedunculopontine nucleus, which regulates locomotion at the supraspinal level.

## Concluding remarks

The editors of this topic really hope that the innovative neural technology as introduced here is effectively implemented in clinical practice to help patients with neuro-psychiatric disorders.

## Author contributions

GZ launched the topic, drafted the Editorial. TH handled six contributions of the topic, critically and constructively revised the Editorial.

### Conflict of interest statement

The authors declare that the research was conducted in the absence of any commercial or financial relationships that could be construed as a potential conflict of interest.
